# Antioxidant Strategies in the Management of Diabetic Neuropathy

**DOI:** 10.1155/2015/515042

**Published:** 2015-03-02

**Authors:** Ayodeji Babatunde Oyenihi, Ademola Olabode Ayeleso, Emmanuel Mukwevho, Bubuya Masola

**Affiliations:** ^1^Discipline of Biochemistry, School of Life Sciences, University of KwaZulu-Natal, Westville Campus, Private Bag X54001, University Road, Durban 4000, South Africa; ^2^Department of Biochemistry, University of Johannesburg, P.O. Box 524, Auckland Park 2002, South Africa

## Abstract

Chronic hyperglycaemia (an abnormally high glucose concentration in the blood) resulting from defects in insulin secretion/action, or both, is the major hallmark of diabetes in which it is known to be involved in the progression of the condition to different complications that include diabetic neuropathy. Diabetic neuropathy (diabetes-induced nerve damage) is the most common diabetic complication and can be devastating because it can lead to disability. There is an increasing body of evidence associating diabetic neuropathy with oxidative stress. Oxidative stress results from the production of oxygen free radicals in the body in excess of its ability to eliminate them by antioxidant activity. Antioxidants have different mechanisms and sites of actions by which they exert their biochemical effects and ameliorate nerve dysfunction in diabetes by acting directly against oxidative damage. This review will examine different strategies for managing diabetic neuropathy which rely on exogenous antioxidants.

## 1. Introduction

Diabetes refers to a metabolic disorder characterized by relative or absolute deficiency of insulin secretion and/or insulin resistance. The disorder presents a major health problem that currently affects 382 million people around the world including 316 million patients with impaired glucose tolerance. This population may double by 2030 [1]. Diabetes is known to be one of the foremost causes of mortality and morbidity in the world [2]. It affects the quality of patient's life with a variety of symptoms which include pain, weakness, ataxia, impotence, and sensory loss [3]. It is a complex and progressive disease that results in multiple complications which include retinopathy, nephropathy, cardiomyopathy, hepatopathy, and neuropathy [4]. Uncontrolled chronic hyperglycaemia resulting from absolute insulin deficiency (type 1 diabetes) or insulin resistance with or without insulin deficiency (type 2 diabetes) is one of the primary causes of diabetic complications in a number of organs [5]. Type 1 diabetes mellitus is caused by cell-specific autoimmune destruction of the insulin producing beta cells in the pancreas [6]. Type 2 diabetes occurs as a result of the failure of beta cells to compensate for insulin resistance [7] or selective loss of pancreatic beta cells due to viral infections or toxic damage leading to insulin insufficiency.

Hyperglycaemia-induced oxidative and nitrosative stress has been singled out as one of the major links between diabetes and diabetic complications [8]. Hyperglycaemia leads to generation of free radicals due to autoxidation of glucose and glycosylation of proteins [9]. The persistent increase in reactive oxygen species (ROS) and reactive nitrogen species (RNS) accompanied by a decrease in antioxidant activity leads to the occurrence of oxidative and nitrosative stress which can cause endothelial dysfunction, insulin resistance, and alterations in number and functions of pancreatic *β* cells and eventually leads to diabetic microvascular and macrovascular complications [10]. Once ROS and RNS are produced in excess, they cause the structural deterioration of macromolecules (carbohydrates, proteins, lipids, and DNA) leading to their instability and consequently loss of function [11]. ROS and RNS have also been reported to induce several cellular signaling cascades that ultimately lead to the transcription of stress-related genes which promote the development of diabetic complications [12]. NF-*κ*B (nuclear factor kappa-light-chain-enhancer of activated B cells), a nuclear transcription factor, is activated by an elevation in ROS resulting in the transcription of proinflammatory proteins that exacerbates the conditions of the disease. Proinflammatory chemokines and cytokines like macrophage chemotactic protein (MCP-1), tumor necrosis factor (TNF-*α*), and interleukins (IL-1*β* and 6) have been recently implicated in the progression of diabetes to diabetic complications [12]. Hyperglycaemia-induced elevations of ROS have also been reported to be capable of inducing apoptosis in tissues. The Bax-caspase pathway of apoptosis can be activated by ROS leading to a reduction in the electrochemical gradient across the mitochondrial membrane causing a leakage of mitochondrial cytochrome c into cytoplasm that activates caspases leading to apoptosis [13].

Diabetic neuropathy (DN) seems to be the most common and least understood complication being present in over 50% of chronic diabetics [14, 15]. In the United States, DN is the leading cause of diabetes-related hospital admissions and nontraumatic amputation [16]. It can be found late in type 1 diabetes but early in type 2 diabetes and the cause of this occurrence is still not clear [17]. Increased free-radical formation and/or a defect in antioxidant defenses which result in oxidative stress have been implicated in the pathogenesis of diabetic neuropathy [18]. Diabetic neuropathies are heterogeneous and affect different parts of the nervous system with various clinical manifestations [16].

Antioxidants are available endogenously as a normal defense mechanism of the cell or obtained exogenously from diet. Examples include enzymatic antioxidants like superoxide dismutase (SOD), catalase (CAT), glutathione S-transferase (GST), glutathione peroxidase (GPx), and nonenzymatic antioxidants like reduced glutathione (GSH), uric acid, carotenoids, flavonoids, lipoic acid, and vitamins A, C, and E. SOD dismutates superoxide anion (^•^O_2_^−^) to form hydrogen peroxide which is acted upon by CAT and GPx to produce water. GST converts reactive electrophilic species to hydrophilic forms that are easily excretable products as a result of their conjugation with GSH. Vitamins C and E and lipoic acid are involved in the termination of the lipid peroxidation process [19]. The abilities of flavonoids to scavenge free radicals have also been reported [20]. Some specialized proteins also function as antioxidants such as peroxiredoxins, thioredoxins, and glutaredoxins [21]. This review focuses on the various ways by which exogenous antioxidants exhibit their antidiabetic effects on diabetes and its complications especially diabetic neuropathy.

## 2. Overview of Oxidative Stress and Diabetes

Oxidative stress occurs when the rate of production of reactive oxygen and nitrogen species in a cell far exceeds their rate of utilization and conversion to more stable products leading to cellular and tissue damage. The imbalance of prooxidants/antioxidant ratio favouring the former causes an alteration in the normal redox signaling of the cell triggering impairment in several pathways of the cell's metabolism, a critical feature in diabetes [13]. Reactive oxygen and nitrogen species are highly unstable species which are free radical or non-free radical compounds that can be either useful or harmful to the cell. Examples of ROS include free radicals such as superoxide (^•^O_2_^−^), hydroxyl (^•^HO), peroxyl (^•^RO_2_^−^), hydroperoxyl (^•^HRO_2_^−^), and nonradical species such as hydrogen peroxide (H_2_O_2_) and hydrochlorous acid (HOCl). RNS include free radicals like nitric oxide (^•^NO^−^) and nitrogen dioxide (^•^NO_2_^−^) and nonradicals species such as peroxynitrite (ONOO), nitrous oxide (HNO_2_), and alkyl peroxynitrates (RONOO) [19].

Increases in biomarkers of oxidative stress related to lipid (thiobarbituric acid reactive substances (TBARS), malondialdehydes (MDA), and isoprostanes), protein (protein carbonyls and nitrosylated proteins), carbohydrate (advanced glycated end-products (AGEs)), and DNA (8-hydroxy-deoxyguanine (8-OHdG)) together with inhibition of the synthesis of endogenous antioxidants have been observed in several* in vitro* and* in vivo* experimental models of diabetes [22–24]. Hyperglycemia-induced oxidative stress has been reported to inhibit the secretion of insulin in pancreatic beta cell through the activation of an uncoupling protein-2 (UCP-2) which lowers the ATP/ADP ratio by leaking protons in the *β* cell [25]. ROS has been shown to leak into cell membranes and damage pancreatic *β* cells [26, 27]. Overproduction of free radicals like superoxide anion in *β* cells can also lead to the activation of stress-signaling pathways that can induce downstream effectors like NF-*κ*B leading to *β* cell apoptosis and dysfunction ultimately reducing insulin secretion [28, 29].

An experimental study to confirm the effects of high glucose-induced oxidative stress on the pancreatic *β* cells and insulin secretion showed that concentrations of insulin mRNA, insulin content, and insulin release were significantly reduced upon exposure to high glucose [30]. One of the mechanisms of insulin resistance is altered insulin signaling. Insulin signaling is initiated by the activation of a specific insulin receptor. Upon binding of the insulin molecule to *α* subunit of the receptor, the inhibition of tyrosine autophosphorylation by the *β* subunit is released. The activated insulin receptor directly phosphorylates insulin receptor substrates (IRS-1-4) on multiple tyrosine residues. Tyrosine phosphorylated IRS proteins then act as a binding site for a variety of signaling molecules that eventually mediate the release and activation of insulin [31–33]. However, in conditions of increased oxidative stress, stress-responsive signaling cascades are activated leading to the modification of IRS proteins by increased serine/threonine phosphorylation which are subsequently degraded contributing to insulin resistance [34]. High concentrations of hydrogen peroxide (H_2_O_2_) have been shown to directly induce insulin signaling (phosphatidylinositol-3-kinase dependent pathway) leading to insulin resistance prior to the onset of diabetes [34, 35]. ROS have been reported in several studies as performing an important role in insulin resistance in type 2 diabetes and obesity experimental models [36, 37].

Hyperlipidaemia (abnormal increase in lipid levels) in the presence of hyperglycaemia generates additional ROS that are also implicated in *β* cell dysfunction [38]. Excess free fatty acids have previously been shown to cause ROS overproduction leading to mitochondrial DNA damage and pancreatic *β* cell malfunctioning [39]. Mitochondria play a critical role in regulating the metabolic imbalance seen in diabetes-induced oxidative stress since it is the organelle responsible for maintaining the transfer of electrons through the electron transport chain to molecular oxygen during aerobic respiration in cells. This becomes a potential site for the overproduction of reactive species like H_2_O_2_ and ONOO– which can cross mitochondria membranes and damage macromolecules in other cellular regions [40]. Also, ^•^O_2_^−^ levels have been reported to increase in the mitochondrial electron transport chain (ETC) as a result of hyperglycaemia during diabetes leading to an increase in oxidative stress [41]. Other pathways like synthesis of metabolites (through xanthine oxidase pathway), production of neurotransmitters and serotonin, and detoxification of xenobiotics via cytochrome P450 system and NADPH oxidase utilize oxygen molecules with the possibility of ROS formation which add to the burden of oxidative stress in diabetes [42].

## 3. Diabetic Neuropathy (DN) and Oxidative Stress

Several microvascular and macrovascular complications arise as a result of the onset and progression of diabetes [43]. These complications affect the eyes (retinopathy), kidneys (nephropathy), nerves (neuropathy), or heart (cardiovascular diseases) and are mainly responsible for the increase in morbidity and mortality of diabetics worldwide [44]. DN results from peripheral nerve dysfunctions involving different parts of the somatic and autonomic nervous systems which are the basis for many classifications of the disease [45]. Diabetic peripheral neuropathy (DPN) generally encompasses polyneuropathies and some rare varieties which can be further subdivided based on differences in onset, duration, clinical manifestations, and pathophysiology [16, 46].

**Figure 1:**
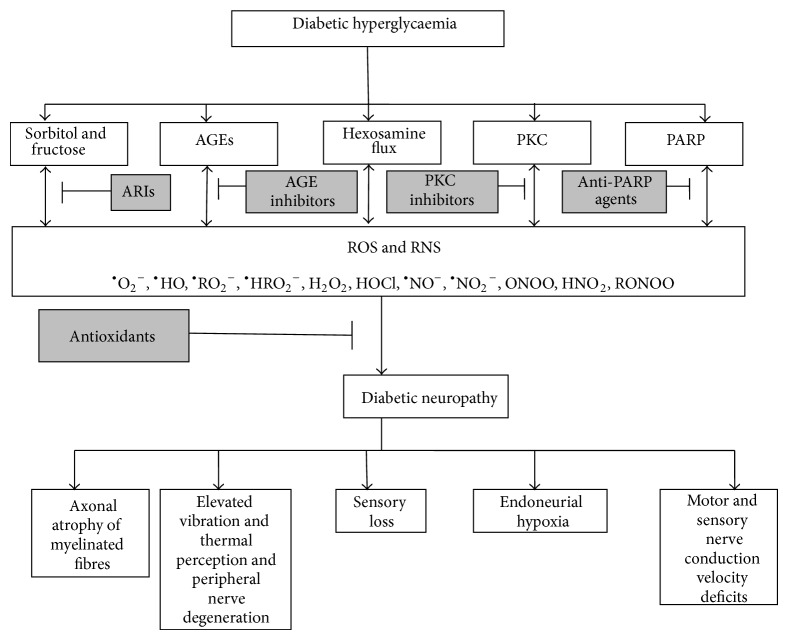
A simplified scheme showing the roles of reactive species and antioxidants in the progression of diabetic neuropathy. AGEs: advanced glucose end-products; PKC: protein kinase C; PARP: poly-ADP ribose polymerase; ARIs: aldose reductase inhibitors; ROS: reactive oxygen species; RNS: reactive nitrogen species; ^•^O_2_^−^: superoxide radical; ^•^HO: hydroxyl radical; ^•^RO_2_^−^: peroxyl radical; ^•^HRO_2_^−^: hydroperoxyl radical; H_2_O_2_: hydrogen peroxide; HOCl: hydrochlorous acid; ^•^NO^−^: nitric oxide radical; ^•^NO_2_^−^: nitrogen dioxide radical; ONOO: peroxynitrite; HNO_2_: nitrous oxide; RONOO: alkyl peroxynitrates.

Oxidative stress (Figure 1) has been implicated in causing nerve damage in several animal, human, and experimental models of diabetes [47–51]. The mechanisms involved in oxidative stress-induced nerve dysfunctions include generation of reactive oxygen species, increased reactive nitrogen species, lipid peroxidation [52, 53], DNA damage, and reduction in cellular antioxidants [48, 54]. Increased reactive oxygen and nitrogen species are capable of damaging lipids present in the myelinated structures of nerves resulting in the loss of axons and disruption of the microvasculature in the peripheral nervous system [45]. Oxidative damage to peripheral nerves causes hyperexcitability in the afferent nociceptors and central neurons leading to the generation of spontaneous impulses within the axons and dorsal root ganglions of the nerves contributing to the neuropathic pain associated with diabetic neuropathy [55]. Recent findings implicate free radicals in the development of diabetic neuropathy in addition to the impairment of antioxidant defense system in type 2 diabetes mellitus patients [15].

High glucose was shown to cause an increase in superoxide anion and peroxynitrite ion, which can damage nerves in diabetic neuropathy [18]. Experimental studies revealed that high glucose induces apoptosis via a mitochondria-dependent route in embryonic sensory neurons [56]. Hyperglycaemia has been postulated to generate oxidative stress via several well-studied, interconnected pathways which ultimately lead to nerve dysfunction essentially by the activation of downstream signaling pathways involving NF-*κ*B, mitogen activated protein kinases (MAPK), proinflammatory cytokines, and gene transcriptions [11]. Some of the pathways of hyperglycaemia-induced oxidative stress include glucose autoxidation, advanced glycation end-products (AGEs) overproduction, increased hexosamine flux, activation of diacylglycerol and protein kinase C, and activation of polyol pathway [11, 57].

### 3.1. Polyol Pathway

**Figure 2:**
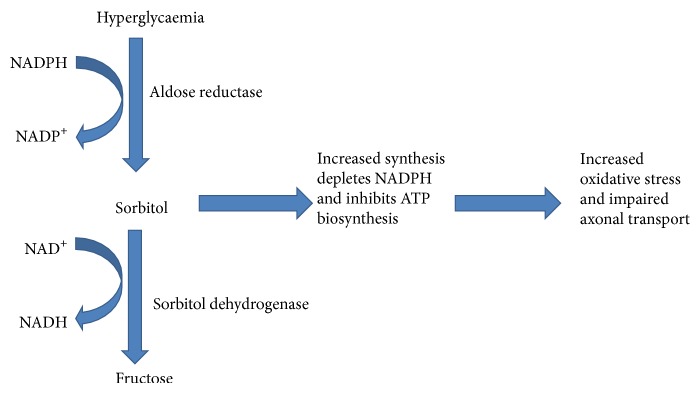
Polyol pathway of hyperglycaemia-induced neuropathy.

Most of the glucose that enters a cell is metabolized via glycolysis to give pyruvate; only about 3% is converted to sorbitol through the polyol pathway. However, in hyperglycaemic conditions such as diabetes, there is an increased flux of glucose into the nerves. Whenever glucose becomes excess, it leads to the saturation of the glycolytic pathway which subsequently increases the activity of the polyol pathway to about 30%. The catalytic actions of aldose reductase and sorbitol dehydrogenase convert the extra glucose to sorbitol and fructose (Figure 2). Since sorbitol cannot cross cell membranes, it accumulates in cells causing hyperosmolarity and concomitant efflux of taurine, myoinositol, and adenosine. This inhibits the biosynthesis of ATP resulting in reduced activity of Na^+^/K^+^ ATPase and protein kinase C (PKC), impaired axonal transport, and structural breakdown of nerves. Also, induction of aldose reductase enzyme depletes NADPH, a requirement for the regeneration of the cellular antioxidant, reduced glutathione, contributing to oxidative stress [11, 46, 58, 59]. Ho and colleagues reported that the peripheral nerves of diabetic mice deficient in aldose reductase showed reduced oxidative stress when compared to diabetic mice possessing the enzyme thus verifying the importance of the polyol pathway in the pathogenesis of acute diabetic neuropathy [54]. Increased sorbitol pathway activity also leads to impaired neurotrophic support [60].

### 3.2. The AGEs Concept

Under hyperglycaemic conditions, the primary amino group of protein reacts nonenzymatically with the carbonyl group of glucose forming Schiff base intermediates through the Maillard reaction. The rearrangements of these intermediates yield Amadori products. Further intra- and intermolecular cross-linking reactions with proteins, lipids, or DNA lead to the formation of stable, covalent, and irreversible adducts collectively referred to as advanced glucose end-products (AGEs) that accumulate within cells with age [11, 57]. Increased formation of AGEs leads to the elevation of oxidative stress and subsequently damage to cells and tissues, an occurrence that has been found in experimental animals and in humans [61–63]. AGEs have also been shown to decrease axonal transport within neurons leading to their degeneration [64]. Similarly, AGEs can bind to RAGE (receptor for advanced glycated end-products) activating it and triggering several downstream signaling and inflammatory pathways ultimately contributing to oxidative stress. AGEs-RAGE interaction elevates oxidative stress through NADPH oxidase activation, NF*κ*B gene expression, and the induction of proinflammatory cytokines activities [13]. This affects the structural integrity of the neurons and disturbs nerve blood flow and hence nerve dysfunction in diabetic neuropathy [46, 65].

### 3.3. Glucose Autoxidation

The first evidence of the role of glucose autoxidation in diabetes was reported by Wolff and Dean [66]. In an environment where hyperglycaemia is prevalent, excess glucose can undergo enediol rearrangement to form an enediol radical which is capable of reducing molecular oxygen to form superoxide anion, a potent radical implicated in the pathogenesis of diabetes. The enediol radical can also form AGEs directly by modifying lysine or arginine amino residues in proteins through the help of transition metal-catalyzed autoxidation. Glucose can also generate ^•^HO radicals which also contribute to the elevation of prooxidants that can attack DNA forming stable covalent adducts that are damaging to the cell [67].

### 3.4. Hexosamine Flux

Fructose-6-phosphate is an intermediate of the glycolytic pathway which is formed from glucose-6-phosphate by the enzyme phosphoglucoisomerase. However, in the presence of high glucose, fructose-6-phosphate can accumulate, and it is utilized by the hexosamine pathway. Here, fructose-6-phosphate is converted to glucosamine-6-phosphate by catalytic action of the enzyme glutamine-fructose-6-phosphate aminotransferase (GFAT). Glucosamine is well documented to increase oxidative stress in cells via the production of H_2_O_2_ [68]. Glucosamine-6-phosphate is further processed via conjugation reactions with uridine triphosphate (UTP) to yield uridine diphosphate-N-acetylglucosamine (UDPGlcNAc). UDPGlcNAc thus formed can attach to the amino group of serine and threonine residues of proteins relevant to the elevation of transcription factor SpI which in turn activates the transcription of growth factors like TGF*α* and TGF*β*1 and plasminogen activator inhibitor-1 (PAI-1) [69]. These proteins are involved in the pathogenesis of diabetes-induced vascular complications especially in the nerve [46, 70]. Similarly, GFAT enzyme has been implicated in insulin resistance and hyperinsulinaemia in type 2 diabetes mellitus [51].

### 3.5. PKC Activation

**Figure 3:**
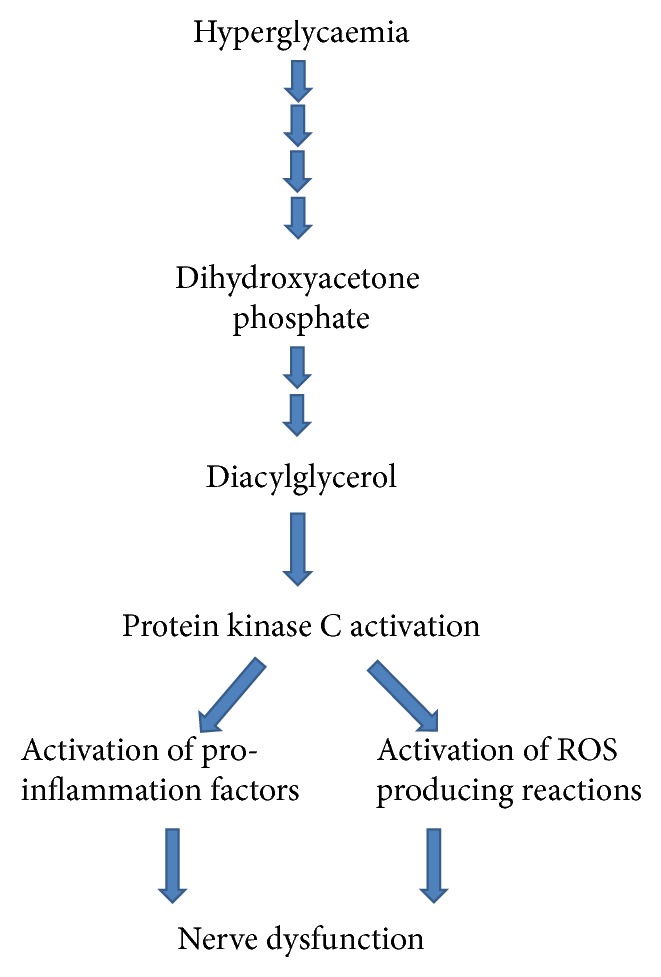
Hyperglycaemia-induced overactivation of protein kinase c leads to nerve dysfunction.

Excess glucose in the intracellular medium results in the accumulation of an intermediate of the glycolytic pathway, dihydroxyacetone phosphate (Figure 3). This leads to the formation of glycerol-3-phosphate which upon conjugation with fatty acids yields diacylglycerol (DAG). DAG is the most important activator of 9 isoforms out of 11 of protein kinase C (PKC) although AGE-RAGE interaction has also been shown to activate it [71]. PKC activation is relevant to nerve function and the pathogenesis of diabetic neuropathy probably through triggering an intracellular signaling cascade resulting in the elevation of the expression of transcription factors like NF-*κ*B, proinflammatory cytokines like transforming growth activator beta (TGF*β*), blood clotting inhibitors like plasminogen activator inhibitor (PAI), and extracellular matrix proteins [72, 73]. PKC has been reported to promote vascular endothelial cell proliferation by activating phospholipase A_2_ and stabilizing vascular endothelial growth factor (VEGF) mRNA expression [72, 73]. The activation of PKC also induces the overproduction of ROS and AGEs by the NADPH oxidase system causing deleterious effects to the cell [74]. PKC can be structurally regulated depending on the redox status of the cell; increased oxidants bind to the regulatory domain promoting its activity while elevated reductants bind to the catalytic domain inhibiting its activity [46]. PKC activation has been suggested to play dual roles in diabetic neuropathy, altering nerve conduction by restricting blood flow when its activity is low or causing impairment of nerve functions by affecting the activity of neurochemicals when its own activity is high [51].

### 3.6. Other Pathways of Hyperglycemia-Induced Oxidative Stress

In addition to the aforementioned pathways, hyperglycaemia-induced oxidative stress also triggers other multiple, interconnected signal transduction cascades including poly-ADP ribose polymerase (PARP) induction [75, 76], mitogen activated protein kinase (MAPK) overactivation [77, 78], calcium signaling [79], growth factors induction, phosphoinositide pathway, and stimulating the enzymes of arachidonic acid metabolism [80–82] which are all involved in the pathogenesis of diabetic neuropathy. The different pathways all seem to have a central recurring effect of oxidative stress in diabetes. Increased ROS and RNS together with significant reductions in the antioxidant defense mechanisms within the neurons contribute to the manifestations of diabetic neuropathy which include nerve blood flow impairment, endoneurial hypoxia, motor and sensory nerve conduction impairment, peripheral nerve degeneration, increased vibration and thermal perception, sensory loss, axonal atrophy of large myelinated fibers, and neuropathic pain.

## 4. Antioxidants and Diabetic Neuropathy

The important roles played by oxidative stress in mediating diabetic neuropathy (DN) cannot be overemphasized and hence it is not surprising to note that antioxidants have occupied the mainstream in the search for an efficient and efficacious treatment of nerve dysfunction in diabetes within the past decade. An increasingly large number of antioxidants and antioxidant-mimicking agents have been tested* in vivo* and* in vitro* in animal experimental models [83–89]. Examples of antioxidants noteworthy of mention are vitamins A, C, and E, curcumin, *α*-lipoic acid, melatonin, acetyl-L-carnitine, and flavonoids. Among antioxidants that have progressed to human clinical trials, few are currently at different stages of evaluation while others have been withdrawn from the study due to lack of efficacy or safety concerns [11]. At present, no antioxidant treatment has been approved by the United States Food and Drug Administration for DN although *α*-lipoic acid, which seems to be the leading antioxidant in clinical trials, has been approved in some European countries [11, 90–92].

## 5. Antioxidant Strategies in Diabetic Neuropathy

Generally, antioxidants work to achieve two main goals: reduce the harmful effects of free radicals either by preventing their formation or by scavenging and inactivating them or boost the natural defense systems by inducing the activities of antioxidant enzymes and regenerating other proteins involved in antioxidant pathways. However, there are several strategies employed in the use of different antioxidants to combat nerve dysfunction in diabetes. The choice of strategy depends on the type, structure, and concentration of the antioxidants. Also, the stage, severity, prevalence, and primary causes of the disease are equally important. Some of the strategies are summarized below.

### 5.1. Strategies Targeted Directly against ROS and RNS

Diabetes-induced nerve dysfunction is established to be caused by an increase in the overproduction of ROS and RNS. The mechanisms involved have been discussed in detail in the previous sections. The main proof of oxidative stress involvement in DN was the discovery that excess free radicals were produced in DN experimental animal models and that there was a reduction in the activities of endogenous antioxidant enzymes, and these effects were ameliorated upon treatment with antioxidant correlating with the alleviation of symptoms of DN [83, 84, 93, 94]. It was therefore hypothesized that antioxidants or agents that directly scavenge free radicals can reduce the formation or progression of ROS reactions which in turn decreases oxidative stress thereby improving DN conditions. Based on these preclinical studies, clinical trials were embarked on to test some novel antioxidants in humans. However, there have been disparities between results obtained from animal and human studies, as majority of the antioxidants performed inadequately in clinical trials. Some of the most important antioxidants include alpha-lipoic acid, vitamins A, C, and E, acetyl L-carnitine, taurine, and melatonin.

#### 5.1.1. Alpha-Lipoic Acid (ALA)

Alpha-lipoic acid (ALA) is thought to be the most successful antioxidant in clinical trials. It is the only antioxidant capable of dissolving in both water and fats [95]. ALA can be biosynthesized in plants and animals where it is metabolized to dihydrolipoic acid (DHLA) upon uptake into cells. Both ALA and DHLA are potent free radical scavengers that are also involved in the regeneration of vitamins C and E and oxidized glutathione within the cell [95, 96]. ALA is also a cofactor for a number of mitochondrial enzymes [96]. In experimental models, ALA was reported to decrease lipid peroxidation, reduce oxidative stress, and improve nerve blood flow and distal, sensory, and motor nerve conduction in diabetic animals [97, 98]. The role of ALA in ameliorating the symptoms of DN has been demonstrated in several clinical trials [18, 90–92, 95, 99–103]. ALA is known to reduce oxidative stress by inhibiting hexosamine and AGEs pathways [101]. In a recent report, ALA600SOD (an oral formulation of ALA and superoxide dismutase) improved symptoms and electroneurographic parameters among subjects with DN [104]. These evidences facilitated the licensed use of ALA (600 mg/day) in Germany to treat symptomatic DN [105].

#### 5.1.2. Vitamins A, C, and E

Dietary antioxidant vitamins such as vitamins A, C, and E detoxify free radicals directly and also interact with recycling processes to create reduced forms of the vitamins [106]. Antioxidant vitamins have a number of biological activities such as immune stimulation and prevention of genetic changes by inhibiting DNA damage induced by the reactive oxygen metabolites [107]. Over the past decade, a lot of attention has been given to vitamins C and E because of their free radical scavenging properties. There are several reports on their important roles in protecting cells from oxidative damage [19, 21]. Vitamin E (tocopherols) reacts with hydroxyl radical to form a stabilized phenolic radical which is reduced back to the phenol by ascorbate and NAD(P)H dependent reductase enzymes [19]. Vitamin E has been reported to alleviate symptoms of diabetes and diabetes-induced complications in animals through reduction in oxidative stress biomarkers [108–111]. Niedowicz and Daleke [112] reported that the preventive effect of vitamin E supplementation in diabetic complications is possibly through a decrease in lipid peroxidation.

In clinical trials, vitamin E did not however show a significant relief of the symptoms of microvascular and macrovascular complications despite reducing oxidative stress biomarkers in the subjects [113–117]. The lack of performance of vitamin E may not however be unconnected to the fact that the design of each study was not targeted directly at diabetes end-points such as <7% glycated haemoglobin levels, <130/180 blood pressure, avoiding hypoglycaemic events, and maintaining weights [118] but rather at complications that may have multiple causal factors. Emphasis must therefore be directed at DN to realize its immense benefits. In streptozotocin-induced diabetic rats, vitamin E reduces neuropathic pain by the modulation of oxidative stress in the dorsal root ganglia [119]. There is paucity of information on the role of vitamin C in DN despite evidence that it normalizes sorbitol concentration in the blood [117], scavenges lipid peroxides, and regenerates reduced glutathione in diabetes [120–124]. Similarly, from available literature, there is little information on the role of vitamin A in the management of DN. More research is needed to ascertain the effects of vitamins A, C, and E in diabetes and DN.

#### 5.1.3. Flavonoids

Flavonoids are the largest and the most important group of polyphenolic compounds in plants [125] and are found in fruits, vegetables, grains, bark, roots, stems, flowers, tea, and wine [126]. Flavonoids are made up of several subclasses that can scavenge free radicals and chelate metals [127, 128]. Flavonoids such as proanthocyanidin [129], luteolin [130], hesperidin [131], fisetin [132], epigallocatechin-gallate [133], rutin [134], and quercetin [135] have been shown to possess antioxidant activities which protect against diabetic nephropathy. Other antioxidants are taurine, acetyl L-carnitine, and N-acetylcysteine which have been demonstrated to reduce the progression of DN [11, 46, 105, 130].

### 5.2. Strategies Targeted against Hyperglycemia

Glycaemic control may likely be the most effective treatment to delay the onset and slow the progress of DN [105]. Once glucose levels are returned to normal in the blood, hyperglycemia-induced overproduction of ROS is brought to a halt, ameliorating the deleterious consequences of oxidative stress in neurons. Vitamin E supplementation reduced blood glucose and glycated haemoglobin levels significantly [136, 137] and had a neuroprotective effect on the total myenteric population, without affecting intestinal area or thickness of the intestinal wall or muscular tunic [137]. Flavonoids such as epigallocatechin gallate [138], rutin [139], aspalathin [140], naringerin [141], quercetin and chrysin [130, 142], and diosmin [143] have been reported to have blood glucose lowering effects. Several natural occurring plants and herbal-based products with antioxidant properties have been reported to normalize glucose parameters in experimental models. Nadiq and colleagues have reported the antihyperglycemic property of* Tinospora cordifolia* in animals and also prevention of hyperalgesia in experimental DN probably by reducing oxidative stress and inhibiting the aldose reductase enzyme [144].* Momordica charantia*, a naturally occurring antioxidant and antihyperglycaemic plant, has been reported to prevent neuronal damage in diabetic mice as well as ameliorate DN [145]. Other plants with known antioxidant and antihyperglycaemic properties in traditional folklore are* Allium sativum* [146]*, Artemesia afra* [147, 148],* Prosopis glandulosa* [149],* Aloe vera*,* Camellia sinensis*, and* Ocimum sanctum* [150]. Research should be conducted to select and screen plant-based nutraceuticals in order to isolate the active constituents that can be further processed to find a potent remedy for DN. This approach can actually reduce treatment costs because traditional medicinal plants are believed to be more affordable when compared to their orthodox counterparts.

### 5.3. Strategies Targeted against Individual Oxidative Stress Pathways

The pathways of hyperglycaemia-induced oxidative stress discussed earlier are potential therapeutic targets in DN. Some of the interventions have resulted in specific therapies, for example, aldose reductase inhibitors, PKC inhibitors, and anti-AGE agents.

#### 5.3.1. Aldose Reductase Inhibitors

In the preceding sections we have discussed the importance of aldose reductase enzyme in the accumulation of sorbitol and fructose. Therefore, aldose reductase inhibitors (ARIs) are agents that reduce the flux of glucose into the polyol pathway thereby preventing the harmful effects of excess sorbitol and fructose in neurons. Results from* in vivo* and* in vitro* animal studies highlighted the positive effect of inhibiting aldose reductase on DN [151, 152]. These studies have been the foundation for embarking on several clinical trials with ARIs with antioxidant activities such as Fidarestat (SNK-860) [153], Epalrestat [154, 155], and Ranirestat (AS-3201) [156, 157]. Among the ARIs that have made it to clinical trials, Epalrestat was licensed in Japan while others (e.g., Tolrestat (AY-2773), Zenarestat (FK-366; FR-74366), and Ponalrestat) were withdrawn due to inefficacy or safety concerns [45, 158]. ARIs prevent the progression of DN [159], enhance sural motor and sensory nerve conduction velocities (NCV) [156, 157, 160], and improve wrist and ankle F-wave latency together with alleviating neuropathic pain [154].

#### 5.3.2. PKC Inhibitors

PKC is involved in the activation of key regulatory proteins responsible for nerve function and synthesis of neurotransmitters. Inhibiting PKC was reported to suppress neuropathic pain [161, 162]. Ruboxistaurin, a specific inhibitor of PKC-1b that possesses antioxidant effects, improves nerve conduction velocity (NCV) and endoneurial blood flow in diabetic rats [163]. In clinical trials, Ruboxistaurin reduces the progression of DN [164] but fails to achieve its primary end-points, vibration detection threshold (VDT) and symptoms reduction.

#### 5.3.3. Anti-AGE Agents

Anti-AGE agents prevent the formation and accumulation of AGEs. They also counteract the AGE-RAGE interactions that might aggravate the oxidative stress damage in DN. Examples are Benfotiamine, Aminoguanidine, and Aspirin which are known for their antioxidant properties through the inhibition of AGE formation [58, 165].

Benfotiamine has been reported to increase transketolase enzyme activity which directs AGE substrates to the pentose phosphate pathway resulting in the reduction of hyperglycaemic damage. It also inhibits the increase in UDP-N-acetylglucosamine (UDP-GlcNAc) that induces the hexosamine pathway activity ultimately reducing tissue AGEs [166, 167]. Benfotiamine improves NCV and endoneurial blood flow in diabetic rats [168]. In combination with pyridoxamine and cyanocobalamin, Benfotiamine improves the vibration perception threshold, motor function, and symptom score [169]. Aminoguanidine has been reported to react with 3-deoxyglucosone, a precursor of AGE, thereby trapping the reactive carbonyls and preventing the formation of AGEs although it has been withdrawn from clinical trial as a result of toxicity [170]. Aspirin has been reported to inhibit the production of pentosidine, a cross-linking AGE, by scavenging free radicals and chelating metal ions in collagen incubated with glucose* in vitro *[171]. *γ*-linolenic acid also showed some improvements in neuropathy tests [45].

### 5.4. Strategies Targeted at Mitochondria

It has been demonstrated that excess superoxide anion radicals (^•^O_2_^−^), hydroxyl radicals (^•^HO), and hydrogen peroxide (H_2_O_2_) are produced during the generation of ATP in mitochondria under hyperglycaemic conditions contributing to increased oxidative damage [172, 173]. In the oxidative phosphorylation process, electrons are transferred by electron carriers NADH and FADH_2_ through four complexes in the inner mitochondrial membrane to oxygen which is then reduced to water; up to 4% of the oxygen can be converted to ^•^O_2_^−^ [29]. In a diabetic state, the rate of glycolysis is increased and ^•^O_2_^−^ is generated continuously at complex II in the mitochondria respiratory chain [174]. It has been postulated that the excess generation of ^•^O_2_^−^ may be the initiation process of oxidative stress-induced diabetic complications like diabetic neuropathy through the overactivation of MAPK, PKC, and NAD(P)H oxidase [42].* In vitro* studies on sensory neurons have revealed that high concentrations of glucose promote the mitochondrial-dependent pathway of apoptosis and oxidative stress [56]. Hyperglycaemia has been reported to cause mitochondrial dysfunction in the sensory neurons of streptozotocin-diabetic rats [175, 176]. Also, the mitochondrial electron transport chain activity is altered in the dorsal root ganglion of diabetic rats [44].

The mitochondrion houses the highest concentration of antioxidants in cells emphasizing its importance to the redox status in the human body [177]. The overexpression of endogenous antioxidants like SOD2 [178], peroxiredoxin-3 [179], and peroxiredoxin-5 [180] protected against mitochondrial oxidative damage and myocardial dysfunction. Ernster and colleagues reported that exogenous administration of alpha tocopherol and N-acetylcysteine reduces mitochondrial oxidative damage* in vitro* [181]. In diabetes, coenzyme Q10 (a mitochondrial antioxidant) has been reported to show promising therapeutic potential [182]. However, low bioavailability of these antioxidants in mitochondria* in vivo* has been a problem [183, 184]. To overcome this challenge, antioxidant agents have been developed to target the mitochondria by conjugation to lipophilic cations exploiting the negative membrane potential (about −140 mV) of the organelle [177]. This strategy has been successful using lipophilic cations like triphenylmethylphosphonium (TPMP) conjugated with coenzyme Q10 as MitoQ10 [11, 183, 184] or with vitamin E as MitovitE [185, 186]. Also, TEMPOL (4-hydroxy-2,2,6,6-tetramethylpiperidine-1-oxy radical) as MitoTEMPOL, a potent antioxidant that scavenge ^•^O_2_^−^, has been reported to concentrate in the mitochondria (about 1000-fold) [187, 188] similar to PBN (alpha-phenyl N-tertiary-butyl nitrone) as MitoPBN [189].

Szeto-Schiller (SS) peptides, a novel class of peptides, have the capacity to selectively enter the inner mitochondrial membrane and have been investigated for their antioxidant properties in neurodegenerative diseases [190, 191]. Uncoupler proteins (UCPs) are normally lipophilic weak acids that are capable of lowering the membrane potential gradient and may reduce the production of ^•^O_2_^−^ from the mitochondria. Therefore, agents that induce the activities of endogenous uncouplers (UCPs) or administration of low dose artificial uncouplers may become important therapeutics against mitochondria-derived ROS [172, 192].

NADPH oxidase complex mainly catalyses the transfer of electrons from NADPH to molecular oxygen but also generates ^•^O_2_^−^ and H_2_O_2_ targeted at destroying pathogens and bacteria [193]. Under hyperglycaemic environment, NADPH oxidase produces elevated levels of ROS that can cause mitochondrial dysfunction leading to more generation of ROS thereby forming a cycle of ROS production [177]. NADPH oxidase together with nitric oxide synthase has been reported to increase ^•^O_2_^−^ levels in the blood vessels of type 2 diabetes subjects [194]. High glucose increases ROS via the upregulation of NADPH oxidases in the diabetic kidney vasculature [195].

## 6. Conclusion

There has been lack of a comprehensive review that covers all the current antioxidant strategies used to manage diabetic neuropathy and which includes recent advances in these strategies. This review therefore gives a comprehensive treatment of recent advances in these antioxidant strategies and includes those that have dual antihyperglycaemic/antioxidant end-points. The potential of these strategies in managing DN is also assessed by a review of the results of experiments where such strategies have been employed. These results show the success of different strategies in ameliorating oxidative stress by scavenging oxidants or inhibiting pathways that generate them. Such studies have generally focused on particular end-points; thus, they are not holistic in the end-points they explore for each strategy. Secondly there is little combinational application of strategies although some attempts have been made, this approach seems essential since diabetes mellitus is a heterogeneous disease with multiple aetiologies. Thirdly the progression of these strategies to clinical trials has been limited despite evidence from nonclinical studies showing beneficial effects.

A major characteristic of diabetes is hyperglycaemia which underlies several mechanisms involved in the generation of oxidative stress that eventually leads to DN. Oxidative stress has been implicated in the onset and development of impaired insulin secretion and insulin resistance, the two main mechanisms involved in diabetes. Hyperglycaemia-induced oxidative stress remains the most understood means of progression of diabetes to diabetic neuropathy. Therefore, therapies based on combating hyperglycaemia and oxidative stress may serve as safe, cost-effective solutions in the prevention/treatment of diabetes and diabetic neuropathy.

This may be an opportune time to holistically explore the use of antioxidants in solving the lingering problem of diabetic neuropathy. Two antioxidant strategies may hold the key. First is the administration of traditional antioxidants, for example, vitamins A, C, and E and alpha lipoic acid, that have the capacity to rapidly scavenge a variety of free radicals in animal models and human clinical trials of diabetic neuropathy. Combinational approaches including such components as vitamins A, C, and E, alpha lipoic acid, and medicinal plant products with antihyperglycaemic and antioxidant properties need to be explored. The advantages of such combinations include multiplicity of effects targeting different stages in the progression to DN, natural occurrence with some components being dietary constituents, and a generally low toxic potential. Another important advantage of such a strategy is that a more complete range of end-points can be assessed and for this reason, this strategy should be the focus of clinical trials. Second is the effective delivery of therapeutic doses of antioxidant agents into mitochondria, the most important site for the production of ROS in cells. This strategy may either drastically reduce the concentrations of ^•^O_2_^−^, ^•^HO, and H_2_O_2_ that may initiate oxidative damage to cells or induce the activities of the mitochondria antioxidants to “mop up” the ROS and RNS produced.
